# Nanocarbon-Enhanced 2D Photoelectrodes: A New Paradigm in Photoelectrochemical Water Splitting

**DOI:** 10.1007/s40820-020-00545-8

**Published:** 2020-11-13

**Authors:** Jun Ke, Fan He, Hui Wu, Siliu Lyu, Jie Liu, Bin Yang, Zhongjian Li, Qinghua Zhang, Jian Chen, Lecheng Lei, Yang Hou, Kostya Ostrikov

**Affiliations:** 1 Key Laboratory of Biomass Chemical Engineering of Ministry of Education, College of Chemical and Biological Engineering, Zhejiang University, Hangzhou, 310012 People’s Republic of China; 2 School of Chemistry and Environmental Engineering, Wuhan Institute of Technology, 206 Guanggu 1st Road, Wuhan, 430205 People’s Republic of China; 3 Department of Environmental Science and Engineering, North China Electric Power University, 619 Yonghua N St, Baoding, 071003 People’s Republic of China; 4 Institute of Zhejiang University - Quzhou, Quzhou, 324000 People’s Republic of China; 5 Ningbo Research Institute, Zhejiang University, Hangzhou, 315100 People’s Republic of China; 6 State Key Laboratory of Industrial Control Technology, College of Control Science and Engineering, Zhejiang University, Hangzhou, 310012 People’s Republic of China; 7 School of Chemistry and Physics and Centre for Materials Science, Queensland University of Technology, Brisbane, QLD 4000 Australia

**Keywords:** Advanced nanocarbons, Co-catalysts, 2D layered structure, Integrated photoelectrodes, Photoelectrochemical water splitting

## Abstract

Solar-driven photoelectrochemical (PEC) water splitting systems are highly promising for converting solar energy into clean and sustainable chemical energy. In such PEC systems, an integrated photoelectrode incorporates a light harvester for absorbing solar energy, an interlayer for transporting photogenerated charge carriers, and a co-catalyst for triggering redox reactions. Thus, understanding the correlations between the intrinsic structural properties and functions of the photoelectrodes is crucial. Here we critically examine various 2D layered photoanodes/photocathodes, including graphitic carbon nitrides, transition metal dichalcogenides, layered double hydroxides, layered bismuth oxyhalide nanosheets, and MXenes, combined with advanced nanocarbons (carbon dots, carbon nanotubes, graphene, and graphdiyne) as co-catalysts to assemble integrated photoelectrodes for oxygen evolution/hydrogen evolution reactions. The fundamental principles of PEC water splitting and physicochemical properties of photoelectrodes and the associated catalytic reactions are analyzed. Elaborate strategies for the assembly of 2D photoelectrodes with nanocarbons to enhance the PEC performances are introduced. The mechanisms of interplay of 2D photoelectrodes and nanocarbon co-catalysts are further discussed. The challenges and opportunities in the field are identified to guide future research for maximizing the conversion efficiency of PEC water splitting.

## Highlights

- Layered integrated photoelectrodes for water splitting incorporating nanocarbon co-catalysts are systematically reviewed.
- The correlations between intrinsic structures, optimized configurations, and water splitting performances of layered integrated photoelectrodes are established and analyzed.
- Various synthetic strategies and assembling procedures are critically examined to enhance water splitting performance of layered integrated photoelectrodes.
- Current challenges and future directions for maximizing the efficiency of photoelectrochemical water splitting are outlined.

## Graphical abstract

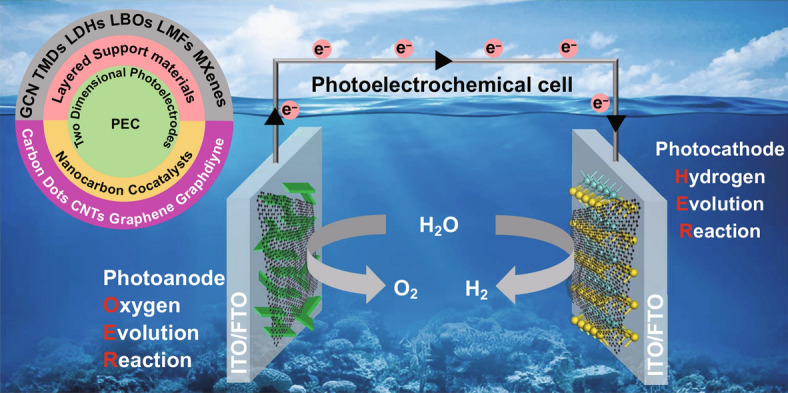

## Introduction

Conversion of clean and renewable solar energy into storable chemical energy is a viable and sustainable approach to solving the grand challenges of exhaustible fossil fuels and environmental pollution. In 1972, it was reported that clean H_2_ energy can be produced from water splitting through a photoelectrochemical (PEC) cell with a TiO_2_ photoanode [1]. What is why combining heterogeneous photocatalysis with electrochemistry is a powerful strategy to achieve the efficient conversion of solar energy [2, 3]. In a typical PEC system, semiconductor-based photoelectrode is the key component, which captures solar light and promotes redox reactions, including water splitting [4, 5], CO_2_ reduction [6, 7], and N_2_ fixation [8, 9].

Upon light irradiation, a semiconductor photoelectrode can be excited by the photons which possess equal or greater energy than its bandgap energy. As a result, photogenerated holes remain in the valence band, while photogenerated electrons are formed and separated within picoseconds, and then jump to the conduction band. The photoinduced holes and electrons are then rapidly transferred to the interface between the photoelectrode and the electrolyte, guided by the intrinsic energy band alignment and the applied electric field [10, 11]. The photogenerated electrons trigger reduction reactions such as hydrogen evolution reaction (HER), CO_2_ reduction, and N_2_ fixation. The corresponding holes in turn take part in oxidation reactions such as the oxygen evolution reaction (OER) and organic pollutant degradation. Importantly, for large-scale applications of solar-driven water splitting, a PEC system is required to possess high energy conversion efficiency, high HER and OER reaction rates, long-term stability, operational safety, and low cost.

Up to now, although major efforts have been made to approach the expected targets, the achieved catalytic performance is still insufficient for practical applications. Therefore, developing advanced high-performance energy materials, and obtaining deep insights into fundamental principles of photochemical conversion and electrocatalytic processes in PEC system are essential to fabricating highly efficient photoelectrodes for solar-driven water splitting [12].

**Fig. 1:**
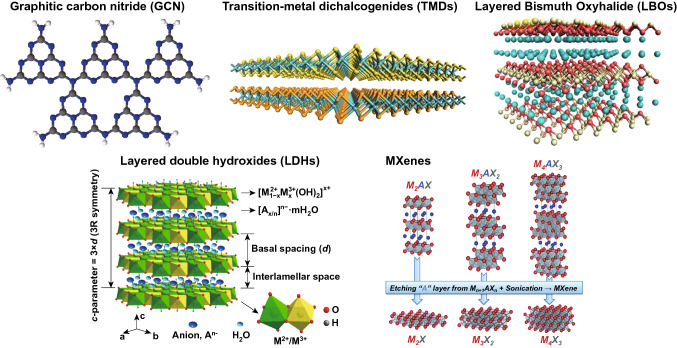
Crystal structures of various 2D layered materials used in PEC solar energy conversion [172, 184–187]. Copyright 2017 and 2016 American Chemical Society, 2015 Royal Society of Chemistry, 2013 Springer Nature and 2017 MDPI (Basel, Switzerland)

Aiming at increasing the overall efficiency for PEC water splitting, advanced two-dimensional (2D) energy materials, including graphitic carbon nitrides (GCNs) [13], transition metal dichalcogenides (TMDs) [14], layered double hydroxides (LDHs) [15], layered bismuth oxyhalides (LBOs) [16], and MXenes [17], etc., have been developed in the past decades, as shown in Fig. 1. Compared with conventional semiconductor photoelectrodes that are usually limited by low accessibility of active sites, 2D layered photoelectrodes feature more active exposed edges and atomic defects, which are beneficial for the PEC water splitting. Experimental and theoretical results reveal that the ultrathin 2D geometry with reduced thickness can provide highly exposed surface area and abundant active atoms. Meanwhile, interior atoms could be brought closer to the surface in thinner atomic layers, thereby facilitating the contact between the catalysts and reactants [18], while more exposed active sites can be utilized for surface catalytic reactions. In addition, rich surface defects, unsaturated atoms, and/or active edges can be produced along with the formation of ultrathin layered structures. Therefore, designing ideal 2D layered photoelectrodes for achieving efficient light capture and charge separation, as well as fast reaction kinetics to increase the PEC water splitting activity, is on the agenda of the forefront research and development efforts.

It is well known that to achieve high-quality photoelectrodes, efficient electrocatalyst/co-catalyst is an inevitable component in a PEC system. Commonly, a proper electrocatalyst can be introduced into a PEC system as a co-catalyst to accelerate photoelectrocatalytic reactions as well as to improve photoinduced charge separation and transportation efficiency at the junctions/interfaces between co-catalyst and light harvesting semiconductor [19–21]. In the past decades, proton reduction co-catalysts (e.g., noble metals [22], metal sulfides [23], metal phosphides [24]), and oxidation co-catalysts (e.g., IrO_*x*_ [25], CoO_*x*_ [26], Co_3_(PO_4_)_2_ [27], CoCuO_*x*_ [28], WO_3_ [29]) have been widely developed for water reduction and water oxidation reactions, respectively. Besides, the unique advantages of strong interfacial interactions between the supporting 2D photoelectrodes and electrocatalysts in an integrated photoelectrode and electrocatalyst system have been recognized [30].

As a class of multifunctional co-catalysts, nanocarbon materials have been widely studied and explored in the past two decades, owing to their high conductivity comparable to metals, which means that free electrons can rapidly move in the whole carbon material [31–34]. For instance, graphene and graphdiyne (GDY) are formed by a single-layer carbon with *sp*^*2*^ bonds and mixture of *sp* and *sp*^*2*^ bonds, respectively. Both these materials possess high charge and mass transport ability [35–38]. Moreover, density functional theory (DFT) calculations reveal that the charge mobility of single-layer graphene and graphdiyne can reach up to ~ 3×10^5^ and 2 × 10^5^ cm^2^ V^−1^ s^−1^, respectively, which infers the excellent electron transport behavior. Subsequently, a series of graphene- and graphdiyne-based electrocatalysts have been developed for HER and OER by means of combining with other host 2D photoelectrodes, including conventional metal oxides and above-mentioned 2D GCNs, TMDs, or LDHs [39–42]. Apart from 2D graphene and graphdiyne, other carbon allotropes, such as carbon nanotubes (CNTs) and carbon dots (CDs), acting as 1D and 0D electron shuttles and collectors, have been utilized to modify 2D photoelectrodes. These integration strategies provide a viable opportunity to explore the 2D PEC systems for solar-driven water splitting in scientific research and engineering fields [43–46].

In this review, the fundamental principles of solar-driven PEC water splitting and critical physiochemical properties of 2D photoelectrode materials are introduced and discussed systematically. We critically analyze the current status of various integrated photoelectrodes made of 2D layered photoelectrodes as host materials, such as GCN, TMDs, LDHs, LBOs, and MXenes, and advanced nanocarbons, such as graphene, graphdiyne, CNTs, and CDs as co-catalysts to achieve highly efficient PEC water splitting. Finally, the critical challenges in this research field are identified to offer our perspective on maximizing the PEC conversion efficiencies of these 2D integrated photoelectrodes for water splitting.

## PEC Water Splitting System

### Components of PEC System

**Fig. 2:**
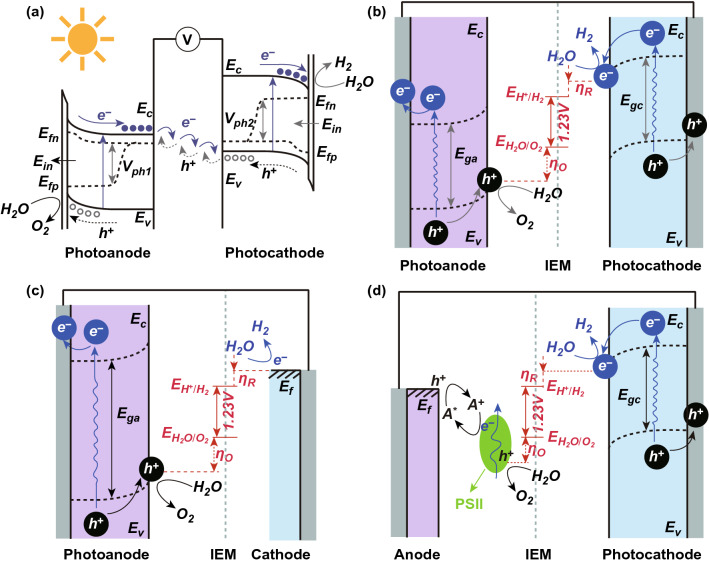
**a** Scheme of energy diagram in a typical PEC system. Ein: built-in electric field in depletion region, Ef: Fermi level, Ec: conduction band, Ev: valence band, Efn: quasi-Fermi level of holes, Efp: quasi-Fermi level of electrons. PEC systems with different models of **b** photoanode and photocathode, **c** photoanode alone, and **d** photoanode along with PSII and A +/A*redox couple. Reproduced with permission from Ref. [47]. Copyright 2015 Wiley–VCH

Commonly, a typical PEC water splitting system is comprised of at least two electrodes partitioned by using an ion exchange membrane, where the oxidation and reduction reactions can be carried out independently on the photoanode and the photocathode, respectively [47]. As presented in Fig. 2a, *n*-type semiconductor acting as a photoanode has the Fermi energy level above the potential of electrocatalytic water splitting, resulting in an upward band bending, upon semiconductor contacting with electrolyte. In contrast, the contact of a *p*-type semiconducting photocathode with electrolyte leads to a downward band bending [48, 49]. Once excited by incident light, host semiconductors are able to absorb photons with higher energies than the bandgap energies of semiconductors and produce photoinduced charge carriers. Subsequently, photogenerated charge carriers are separated at a timescale of picoseconds by built-in electric field (*E*_in_) in the depletion region, thus resulting in the formation of photopotential (*V*_ph1_ or *V*_ph2_) as a driving force for electrocatalytic reactions [50].

Furthermore, owing to different functions of PEC systems, they can be configured with different electrodes and electrolytes for achieving efficient water splitting under light illumination. As shown in Fig. 2b, for a tandem PEC system, it is comprised of a photocathode and a photoanode in single PEC cell, in which both photoelectrodes are used and illuminated simultaneously, and the generated photoelectrons and holes are subsequently participated in the solar-driven overall water splitting [51].

When the photocathode and photoanode are excited by incident photons at the same time, two photopotentials are simultaneously formed and then drive the electrocatalytic redox reactions on the surface [52]. From the thermodynamic viewpoint, the total photoinduced potential (*V*_p_) should be larger than the theoretical redox potential of overall water splitting (*E*_H2O_ = 1.23 V) as unavoidable kinetic overpotentials for HER (*η*_R_) and OER (*η*_O_) need to be overcome when photoelectrodes contact with electrolytes in a practical PEC system. Furthermore, considering the existence of various additional resistances, the potential loss (*η*_other_) across the two-electrode PEC system should be added. Since both photocathode and photoanode can contribute to *V*_p_, many candidate materials with the suitable bandgap energies are used to assemble a tandem PEC system for harvesting sunlight in a broad spectral range and producing strong photocurrent density, thus aiming to achieve high PEC water splitting efficiency.

In contrast, when only a photocathode or a photoanode is involved in a PEC system, a sufficiently high photoinduced potential of the photoelectrode needs to be produced to achieve the overall water splitting. Therefore, the host semiconductor photoelectrode is required to have a wide bandgap (Fig. 2b, c), which is detrimental to the sunlight absorption, thus leading to low photocurrent density and poor solar energy conversion efficiency. To solve this issue, external photosensitizers and co-catalysts need to be introduced and integrated with the wide-bandgap semiconductor to improve light absorption capacity and water splitting activity, respectively. In addition, narrow-bandgap semiconductors (e.g., Si) can also be utilized to assemble an efficient photoelectrode by coupling with light absorber and co-catalyst together, as shown in Fig. 2d [53], where sufficiently high photoinduced potential can be produced for the overall water splitting. The charge exchange between photosystem II (PSII) and photoanode is enabled by the redox couple (A^+^/A^*^) to contribute the electric circuit of PEC system [54, 55]. As a typical example, Domen et al. took advantage of Ru(bpy)_3_^2+/3+^ complex as a redox shuttle to increase the open-circuit potential in *n*-type CdS photoanode/Pt PEC system [56]. Here we note that the one-electron redox reaction of Ru complex made a significant contribution to the high open-circuit potential and short-circuit current under visible light irradiation, in comparison with the onset potential of direct water electrolysis. These findings are beneficial for enhancing both the hydrogen and oxygen evolution reactions.

### Material Requirements

As is well known, the water splitting reaction in PEC system involves multi-step proton and electron transfer processes with relatively slow reaction kinetics. As a result, an overpotential is required to achieve the efficient water splitting reaction [57]. In general, the higher reaction overpotential implies the lower solar energy conversion efficiency. A feasible approach is to introduce active electrocatalysts as co-catalysts into the semiconductor photoelectrode to enhance reaction kinetics and decrease overpotentials for the HER and OER reactions in a PEC system [58]. Notably, many semiconductor-based photoelectrodes suffer from chemical corrosion in solution, thereby leading to unstable operation in applications [59]. Therefore, certain efforts have been made to introduce a protective interlayer to isolate the photoresponsive semiconductor from the electrolyte and promote long-term stability of the integrated photoelectrodes. Consequently, the overall solar conversion efficiency of a PEC system is determined by the photochemical and electrocatalytic processes that take place at the semiconductor interlayer and photoelectrode–electrolyte interfaces, respectively [60].

In a photoelectrode, a semiconductor layer mainly acts as a light absorber to produce photogenerated electrons and holes; subsequently, these generated electrons and holes are separated and transferred to the electrode interface for surface redox reactions. It was reported that the potentials and concentrations of the photogenerated charge carriers are closely related to the PEC redox reactions. A common problem is that only a small number of photogenerated charge carriers near the depletion layer of the semiconductor can be separated and transferred to the electrode interface for driving the water splitting reactions. Therefore, it can be concluded that solar-driven water splitting efficiency of a photoelectrode in a PEC system is determined by the three main factors, including (i) photon absorption capability, (ii) photogenerated charge separation and transfer efficiency, as well as (iii) multi-step surface catalytic reaction rate.

First, the absorption efficiency of the incident light has a strong correlation with the intrinsic extinction coefficient and semiconductor nanostructure, which largely determines the maximum range of the utilizable sunlight spectrum [61–63]. When the semiconductor photoelectrode possesses a suitable bandgap energy for harvesting solar energy, the adequate photoinduced potential can be produced for the surface catalytic reactions.

Second, the separation and transfer efficiency of photoinduced charge carriers mainly depends on the recombination and mobility of photogenerated electrons and holes inside semiconductors [64–66]. If the lifetime and diffusion distance of photoexcited charge carriers are longer and larger, respectively, it is easier to separate and transport the carriers, leading to the higher process efficiencies.

Third, the surface catalytic reaction rate is related to the charge injection efficiency and co-catalysts activity at the electrode–electrolyte interface. Moreover, during these photogenerated charge movement processes, the series resistances involved in the PEC systems will undermine both the photocurrent density and photoinduced potential. Consequently, the required overpotentials inevitably become higher and the solar energy conversion efficiency is reduced.

Essentially, the PEC efficiency of water splitting for a photoelectrode mainly depends on the above-mentioned light harvesting efficiency, charge separation and transfer efficiency, and surface catalytic reaction efficiency. A proper design of light absorbers, interfacial properties, and co-catalysts can effectively enhance the separation efficiency of photogenerated charge carriers and reduce the transportation resistances and overpotentials for catalytic reactions [67–69]. In addition, different synthetic methods and materials configurations, such as semiconductor types, crystal phases, and morphologies, also affect the mentioned efficiencies in a PEC device. Notably, a two-component photoelectrode consists of semiconductor support materials as light absorbers and active materials as co-catalysts. Meanwhile, three or more components can be properly integrated together to fabricate more elaborate and efficient photoelectrodes to further increase the efficiencies of sunlight harvesting, photoinduced charge separation and transportation, and surface catalytic reactions.

These points give a clear guidance for the initial screening of desirable photoelectrode materials and device optimizations at a later stage. Apart from these factors, more convincing and accurate parameters, for instance, onset potential, photocurrent density, incident photon-to-current efficiency (IPCE), and catalyst stability, are required to select the optimal photoelectrode materials.

Furthermore, in order to clearly understand the PEC mechanism and further obtain high water splitting performance, tremendous efforts have been made to experimentally measure the photoinduced charge carrier dynamics, including separation, transportation, and recombination. Various frequency- and time-resolved spectroscopy techniques have been developed, such as electrochemical impedance spectroscopy (EIS), transient photocurrent spectroscopy, and intensity-modulated photocurrent/photovoltage spectroscopy (IMPS/IMVS) [70–72]. Among them, EIS spectroscopy is widely utilized to explore photogenerated charge dynamics and recombination kinetics in the photoelectrodes, where the impedance of a system can be measured over a range of frequencies, thereby facilitating to reveal the frequency response (such as dissipation properties) of the PEC system [73]. Besides, transient absorption spectroscopy (TAS) is an effective tool to obtain absorption and concentration data of photoinduced charge carriers in the PEC system, which is helpful to measure the lifetime of photogenerated electrons and holes, and thereby analyze transport and recombination dynamics in photoelectrodes on the picosecond to microsecond time scales [74–76]. Moreover, time-resolved photoluminescence (TRPL) spectroscopy is also a powerful technique for charge carrier dynamic measurements, which can provide valuable information on photogenerated charge carrier lifetime and electron–hole diffusion length for investigating the size-, surface- and interface-dependent effects [77].

## 2D Photoelectrodes Integrated with Nanocarbons Co-catalysts

### Graphitic Carbon Nitride (GCN)

Metal-free layered GCN has attracted attention as a 2D photocatalyst material owing to its facile synthesis, low cost, and long-term stability. For a PEC system, a suitable bandgap of ~ 2.7 eV with the appropriate band positions makes GCN a promising candidate for water splitting [78–80]. However, the solar-to-energy conversion efficiency of GCN photocatalysts is still far from satisfactory due to the limited light harvesting ability, excessive recombination of photogenerated electron–hole pairs, poor charge carrier transport efficiency, and slow interfacial reaction kinetics in PEC water splitting.

To overcome these weaknesses of the GCN photoelectrodes, introducing nanocarbons as co-catalysts has been widely explored in the past decades. As a 0D carbon material, carbon dots (CDs) with the primary particles sizes of 2–10 nm could be anchored on the surface of GCN via π–π stacking interactions, thus leading to the narrowed bandgap and enhanced light response [81, 82]. Owing to the formation of van der Waals heterojunction between the CDs and GCN, as well as high conductivity of CDs, the separation efficiency of photogenerated charge carries on the CDs/GCN interface could be enhanced [83]. For example, Kang et al. reported that coupling of CDs with porous GCN together via the negative surface charge interaction boosted the quantum efficiencies to the maximum value of 16% at 420 nm with the overall solar energy conversion efficiency of 2.0% under AM 1.5G solar illumination [84]. Besides, Guo et al. fabricated a *p*-CN/CDs hybrid as an integrated photoanode using an electrostatic attraction technique. The photoanode showed an enhanced anodic photocurrent density of 38 μA cm^−2^ at 1.0 V vs. RHE with IPCE of 7.0% at 420 nm under AM 1.5G illumination, which were three and two times higher than the pristine *p*-CN, respectively [85]. The calculated conduction band potential of − 0.59 eV for *p*-CN/CDs demonstrated the desirable thermodynamic merit for PEC-HER.

Compared with the 0D CDs providing only the point-to-point contact in a heterojunction, coupling GCN nanosheets with other carbon materials, such as 2D graphene, can further increase the contact area for fast charge transfer and further improve the PEC conversion efficiency. Recently, a layered CN/reduced graphene oxide (CN-RGO) photoelectrode with rich porous structure was synthesized [86]. In the CN-RGO, the layered heterojunction provided the longer electron diffusion distance of up to 36 μm, higher active surface area, and larger light-response range in comparison with pristine CN. Based on these advantages, the IPCE of the optimized CN-RGO photoanode was impressive 5.3% at 400 nm with a redshift onset wavelength at around 510 nm. Further, the photocurrent density of CN-RGO reached 75 μA cm^−2^ at 1.23 V vs. RHE without any hole scavenging layer, which was 20 times larger than in the pristine CN. Meanwhile, the external quantum efficiency reached 5.5% at 400 nm. Acting as an effective charge transfer bridge, the RGO with excellent conductivity could efficiently accelerate the photoinduced electron transfer from GCN to the substrate, thereby increasing the PEC-HER efficiency. Besides, Leung et al. reported an RGO-modified g-C_3_N_4_/Ni foam photoanode, in which a heterostructure between g-C_3_N_4_ and RGO was formed, thus improving PEC-HER performance [87]. The optimized photoanode of g-C_3_N_4_/RGO (CNG)-Ni foam showed a stable transient photocurrent density of 0.5 mA∙cm^−2^ at 0.4 V vs. SCE with the maximum H_2_ production rate of 6.0 mmol h^−1^ cm^−2^, which were 2.5 and 2.0 times higher than the pristine g-C_3_N_4_, respectively. Under visible light illumination, the photoinduced electrons produced at g-C_3_N_4_ can be rapidly transferred to RGO and Ni foam for H_2_ evolution due to the strong interaction between g-C_3_N_4_ and RGO.

**Fig. 3:**
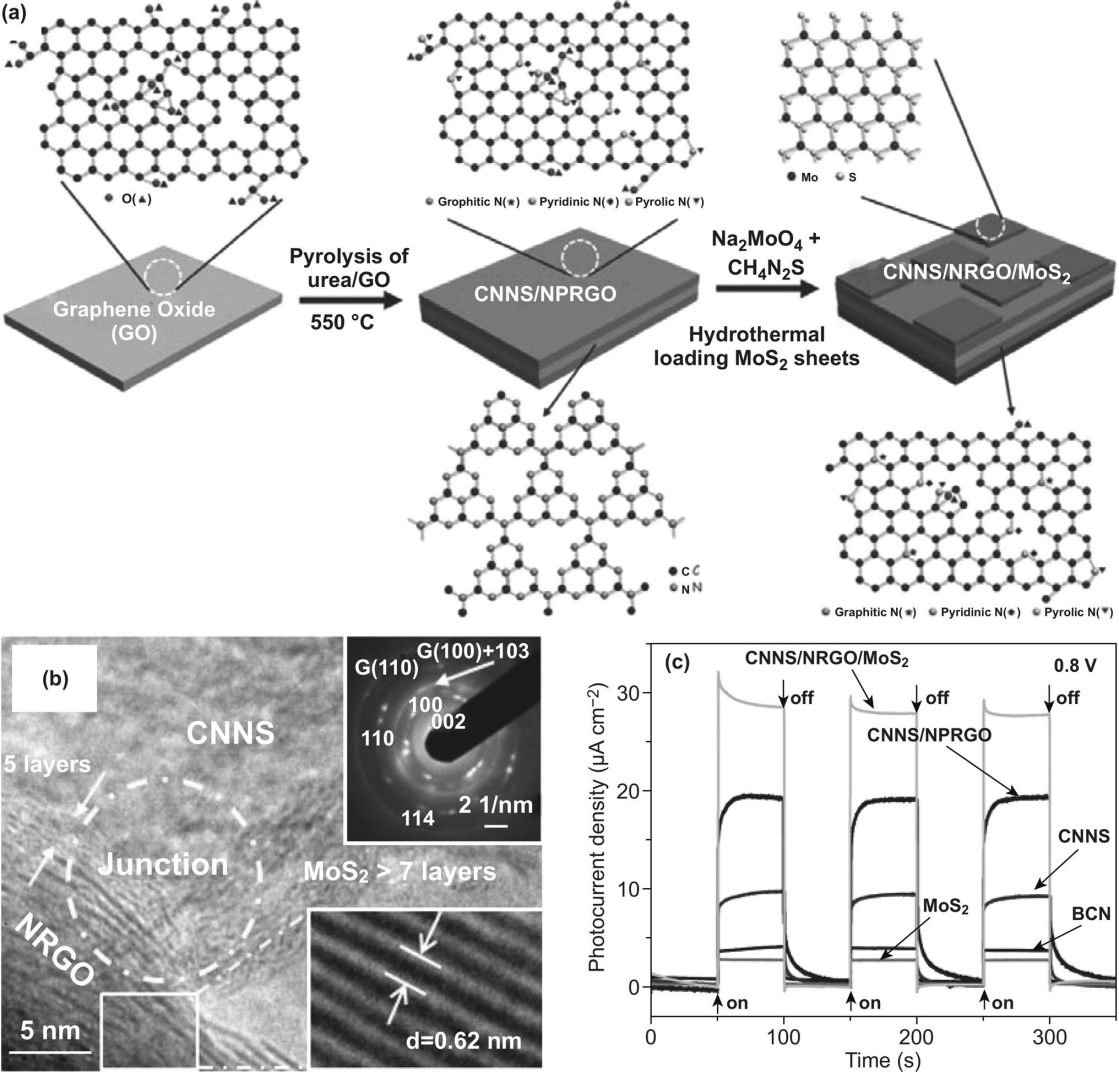
**a** Synthetic scheme of CNNS/NRGO/MoS_2_. **b** HRTEM image of CNNS/NRGO/MoS_2_. **c** Transient photocurrent density vs. time plotted for BCN, MoS_2_, CNNS, CNNS/NPRGO, CNNS/NRGO/MoS_2_. Reproduced with permission from Ref. [88]. Copyright 2013 Wiley–VCH

Apart from conventional 2D GCN/RGO systems, GCN/RGO hybridized heterostructure was also designed and fabricated for efficient PEC water splitting. Recently, a ternary 2D g-C_3_N_4_/N-doped graphene/2D MoS_2_ (CNNS/NRGO/MoS_2_) photoanode was developed via a facile sol–gel deposition route (Fig. 3a, b) [88]. Firstly, the CNNS/NRGO composite was prepared via one-step pyrolysis of urea and GO, where N-containing species were released from polycondensation of urea, resulting in partial reduction and N-doping of GO. Subsequently, the MoS_2_ nanosheets were introduced into CNNS/NRGO hybrid via a hydrothermal treatment to form the CNNS/NRGO/MoS_2_ hybrid. In this unique 2D/2D/2D architecture, the NRGO worked as a charge transfer channel for accelerating electron transfer between g-C_3_N_4_ and MoS_2_, and the g-C_3_N_4_ was used to efficiently harvest sunlight due to its suitable bandgap. Meanwhile, layered MoS_2_ enhanced the light absorption ability, promoted charge carrier separation and transfer across the NRGO/MoS_2_ interface, as well as provided more exposed active sites for PEC-HER. Therefore, such a PEC system was endowed with broadened light harvesting range, shortened charge diffusion distance, and enlarged contact area for the efficient PEC water splitting. As a result, the CNNS/NRGO/MoS_2_ heterojunction exhibited the substantially improved photocurrent density of 37.6 μA cm^−2^ upon loading NRGO, namely 1.46 and 3.43 times higher than that of the two-component CNNS/MoS_2_ (25.7 μA cm^−2^) and CNNS (11 μA cm^−2^) at 0.9 V, respectively, as shown in Fig. 3c.

**Fig. 4:**
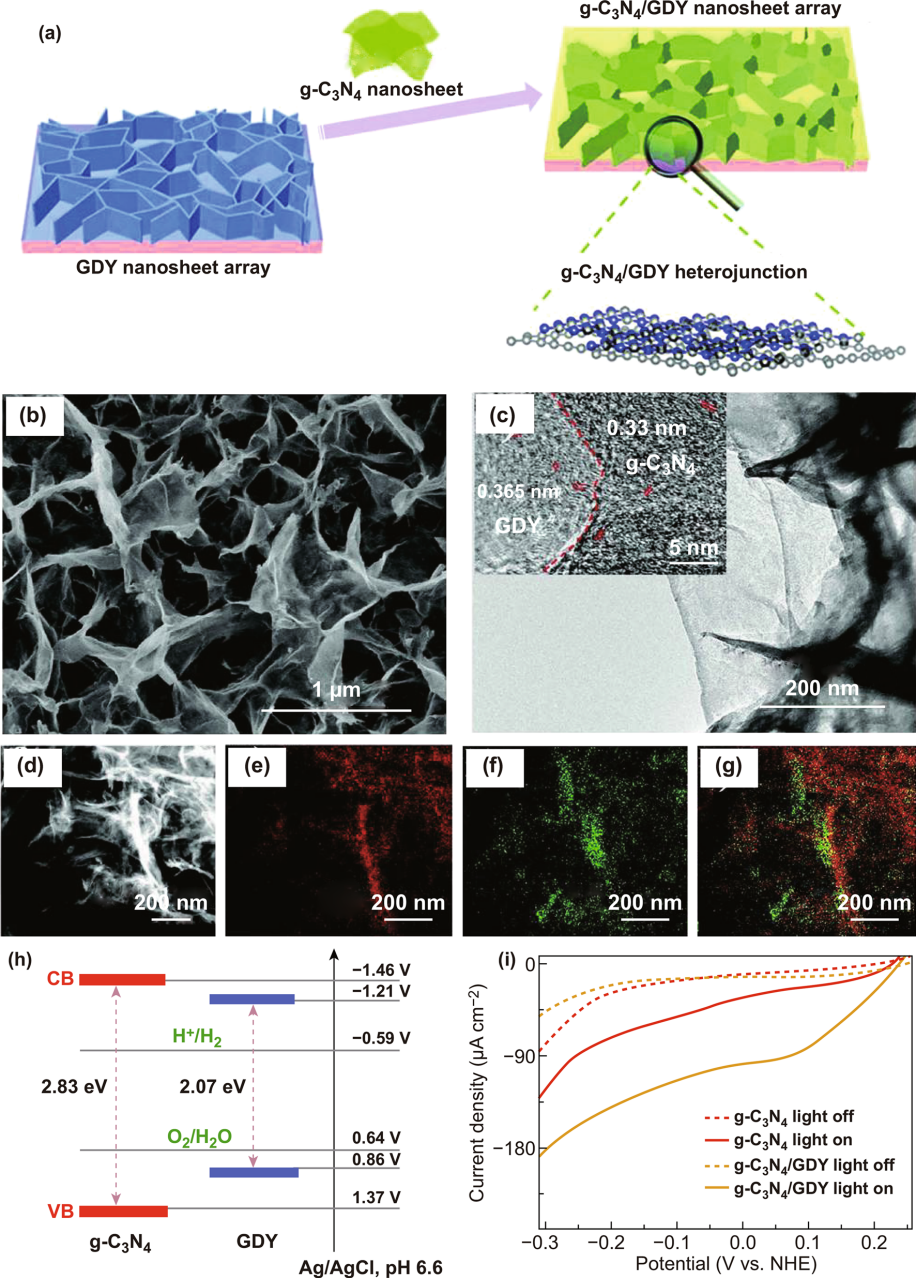
**a** Synthesis scheme of 2D/2D g-C_3_N_4_/GDY; **b** SEM and **c** TEM images of g-C_3_N_4_/GDY; **d** high-angle annular dark field (HAADF) image, **e** C, **f** N, and **g** mixed elemental mapping of g-C_3_N_4_/GDY; **h** band alignments of g-C_3_N_4_ and GDY; **i** linear sweep voltammetry of g-C_3_N_4_/GDY under light on and off. Reproduced with permission from Ref. [92]. Copyright 2018 WILEY

Furthermore, as a unique 2D single-atom-thick carbon allotrope, GDY has attracted great interest in the fields of photo/electro catalysis, solar cells, and batteries. Benefitting from the unique *sp* and *sp*^*2*^ mixture carbon atoms, moderate bandgaps of 0.44-1.47 eV, and high charge carrier mobility of 10^4^–10^5^ cm^2^ V^−1^ s^−1^ [89], the GDY can serve as an efficient modifier to enhance the PEC water splitting performance, such as the previously reported GDY/BiVO_4_ [90] and CdSe QDs/GDY [91]. As a typical example, Lu et al. [92] constructed a g-C_3_N_4_/GDY heterojunction as an excellent photocathode for PEC-HER, as shown in Fig. 4a. The stable 2D/2D g-C_3_N_4_/GDY heterojunction with ultrathin layered structure (Fig. 4b), high charge carrier mobility, and large surface area were beneficial for the efficient transfer of photoinduced holes and electrons, suppressed recombination of photogenerated charge carries, while exposing more active sites. In Fig. 4c, the formed 2D/2D heterojunction in g-C_3_N_4_/GDY hybrid was clearly observed, which could offer large face-to-face interface between g-C_3_N_4_ and GDY, and short distance channels for transportation of photoinduced charge carriers. The layered g-C_3_N_4_ interfaced with the 2D GDY was further verified by EDX elemental mapping in Fig. 4d-g. Owing to the suitable band alignments of g-C_3_N_4_ and GDY (Fig. 4h), the photoinduced holes extracted from g-C_3_N_4_ could effectively transfer to the GDY. Therefore, the photogenerated electrons and holes were sufficiently separated under the external electric field. Meanwhile, the g-C_3_N_4_/GDY hybrid exhibited the sevenfold increase in the electron lifetime (610 μs) and the threefold increase in the photocurrent density (-98 μA cm^−2^ at 0 V vs. NHE) in comparison with the pristine g-C_3_N_4_ (88 μs and − 32 μA cm^−2^) in 0.1 M Na_2_SO_4_ solution, as presented in Fig. 4i, which indicates that the g-C_3_N_4_/GDY photoanode displayed an excellent performance for PEC-HER.

### Transition Metal Dichalcogenides (TMDs)

In comparison with the GCN, layered TMD materials can offer richer active sites and thickness-dependent electronic structures, which is beneficial for the solar energy conversion to produce hydrogen fuel. Generally, TMD materials, including more than 40 kinds of stable 2D materials, can be denoted as MX_2_ (M means transition metal element, and X represents S, Se, and Te), which possess similar structural properties [93]. Their bulk form is mainly made of X-M-X layers, while the neighboring layers are connected by van der Waals interactions. Nevertheless, the electronic properties of TMD materials are closely related with the atomic compositions. For instance, MoS_2_ and MoSe_2_ are semiconductors, while WTe_2_ and VS_2_ are semimetals, and TaS_2_ and NbS_2_ are metals.

Recently, it was found that the atomic layer number of TMDs materials strongly affects their optoelectronic properties. Based on the theoretical and experimental studies, it was reported that monolayer MoS_2_ has a direct bandgap of 1.9 eV, while the corresponding bulk MoS_2_ has an indirect bandgap of 1.3 eV [94]. Furthermore, it was demonstrated that with the decreasing of atomic layer number, the semiconducting 2H-MoS_2_ transforms to metallic 1T-MoS_2_. This feature is beneficial for accelerating the movement of photogenerated charge carriers, thereby injecting more charge carriers into the electrocatalyst–electrolyte interface for catalytic reaction. Recently, Chen et al. reported that the nanostructured MoS_2_ possessed the higher photocurrent density in comparison with bulk MoS_2_ for PEC-HER [95].

The surface defects in nanostructured MoS_2_ also play the key role in determining overall water splitting activity of photoelectrodes. By means of in situ corrosion of bulk MoS_2_, the creation of surface defects led to the decrease in photocatalytic activity, while these produced edge sites in turn enhanced electrocatalytic HER performance. Compared to S atoms with the two-atom coordination, the mono-coordinated S atoms exhibited the higher catalytic activity. However, the S atoms with three-atom coordination located at the basal plane were saturated atoms with no photoactivity. For unsaturated active S atoms, they could strongly bond to H^+^ in a lactic acid solution and easily reduce H^+^ to H_2_ in the presence of photogenerated electrons [96]. It was concluded that the highly active nanosized MoS_2_ with more exposed edges can provide high HER activity in a photochemical/PEC system. Therefore, based on these merits, 2D nanostructured TMDs catalysts were widely fabricated in the past decades as active components to fabricate the integrated photoelectrodes for PEC water splitting [97–101].

**Fig. 5:**
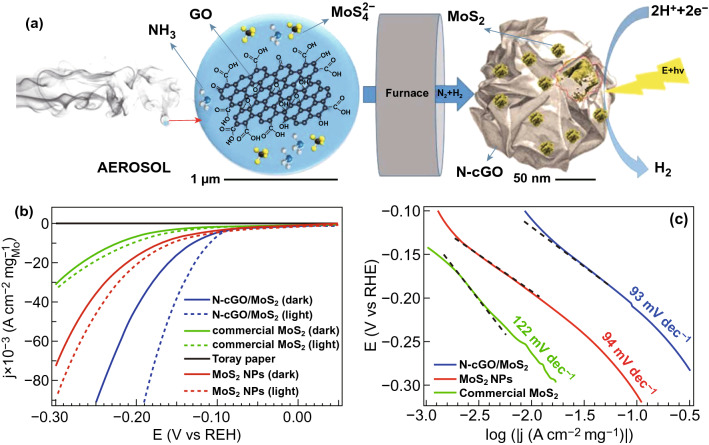
**a** Synthesis scheme of N-cGO/MoS_2_; **b** polarization curves in dark and under illumination and **c** Tafel plots for N-cGO/MoS_2_, MoS_2_ NPs, and commercial MoS_2_ in 0.5 M H_2_SO_4_. Reproduced with permission from Ref. [105]. Copyright 2015 American Chemical Society

However, due to the ultrafast transport of photogenerated charge carriers, the produced electrons and holes are easily recombined, thus decreasing the PEC performance of 2D TMDs photoelectrodes for water splitting [102, 103]. Therefore, to further improve the PEC activity of 2D TMDs-based photoelectrodes, several nanocarbon co-catalysts have been introduced to boost the separation and transfer of photogenerated charge carriers [104]. For instance, Agnoli and co-workers fabricated a *p*-*n* MoS_2_/N-doped cGO heterojunction through an aerosol process in a furnace at 900 °C for enhancing PEC-HER, as shown in Fig. 5a [105]. The N-doped cGO/MoS_2_ hybrid enhanced the HER activity evidenced by the lower overpotential of ~ 100 mV vs. RHE, higher photocurrent density, and smaller Tafel slope with respect to commercial MoS_2_ (Fig. 5b, c). The enhanced PEC-HER activity over the N-doped cGO/MoS_2_ hybrid was mainly caused by the formation of a localized *p*-*n* heterojunction, which facilitated the efficient separation of photogenerated charge carriers.

Subsequently, more attention has been drawn to three-component 2D photocathodes, such as RGO/CdS/MoS_2_ hybrid, for PEC-HER, where the effects of charge transfer behavior and MoS_2_ crystal phase in 2D photocathode on the solar-driven HER were discussed [106]. Owing to the introduction of RGO, the chemical interaction took place between S atoms in CdS/MoS_2_ and C atoms in RGO. The strong electronic interaction between MoS_2_ and RGO was enabled due to the intimate contact, which was beneficial for photoinduced charge separation and transfer with the higher charge density and lower resistance at solid–solid interface, thus boosting the PEC-HER activity.

Besides, nanosized CDs (< 10 nm) have been utilized to modify the TMDs-catalyzed photoelectrodes due to their intrinsically pronounced π-conjugated system, which is beneficial for the rapid electron transfer between TMDs and CDs [107–109]. For example, Kang and co-workers synthesized a CDs decorated MoS_2_ nanosheets via a facile hydrothermal route [110].

The CD-modified MoS_2_ exhibited the significantly enhanced HER ability under visible light irradiation. The mechanism of the improved HER activity of CDs/MoS_2_ can be attributed to the decreased number of S^4+^ and increased amount of S_2_^2−^ and S^2−^ sites, which accelerated the charge carriers transfer between CDs and MoS_2_. Apart from a small Tafel slope of 45 mV dec^−1^ with the reasonably good stability in 0.5 M H_2_SO_4_, the synthesized CDs/MoS_2_ displayed a smaller overpotential of ~ 125 mV at 10 mA cm^−2^ than single MoS_2_. Meanwhile, it was found that the duration of visible light irradiation affected the HER activity, which can be attributed to the extent of reduction in MoS_2_ edges and the formation of surface defects.

**Fig. 6:**
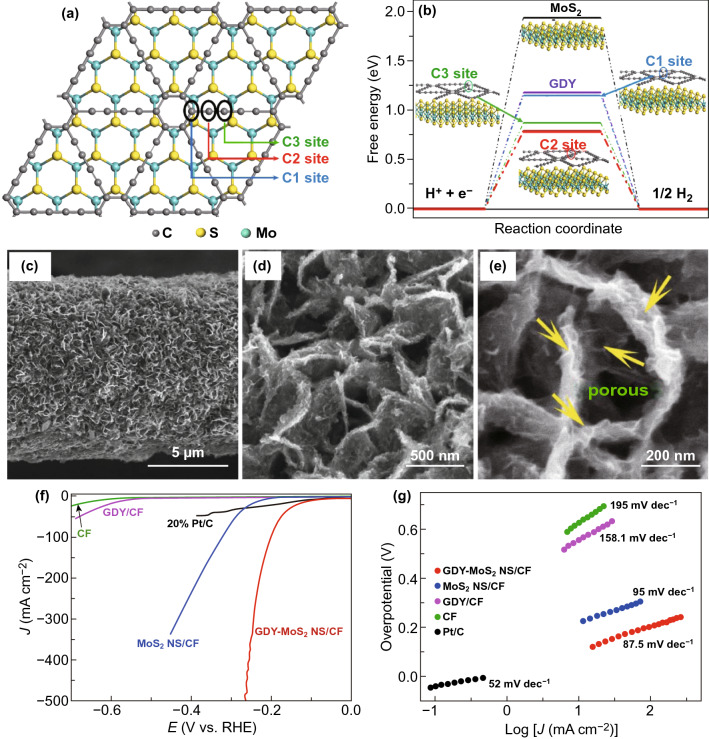
**a** Top view of the optimized GDY-MoS_2_, **b** calculated free energy of HER at equilibrium potential for MoS_2_, GDY and different sites in GDY-MoS_2_ hybrid, **c-e** SEM images of GDY-MoS_2_ NS/CF, **f** linear sweep voltammetry curves, and **g** corresponding Tafel plots of GDY-MoS_2_ NS/CF, MoS_2_ NS/CF, GDY/CF, CF, and Pt/C. Reproduced with permission from Ref. [111]. Copyright 2019 Elsevier

Recently, layered GDY nanosheets have also been utilized to modify the TMD-based photoelectrodes to enhance HER performance. For example, He et al. reported an effective approach to fabricate GDY-coated MoS_2_ nanosheets on a carbon fiber (CF) network for boosting the HER activity under both acidic and alkaline conditions [111]. The theoretical calculations results inferred that the added GDY could effectively anchor on the surface of MoS_2_ nanosheets and result in the transformation of MoS_2_ nanosheets from 2H to 1T phases, which strongly facilitated the transfer of photogenerated charge carriers from MoS_2_ to GDY, as shown in Fig. 6a, b. Upon the coupling of MoS_2_ nanosheets with layered GDY, a hierarchical porous network of GDY-MoS_2_ NS/CF was successfully synthesized, as displayed in Fig. 6c-e, in which the GDY was tightly bonded with MoS_2_ nanosheets with rich porosity, beneficial for improving catalytic activities. Meanwhile, the alkaline PEC tests revealed that the GDY-MoS_2_ NS/CF hybrid had excellent HER activity with a low overpotential of 90 mV at 10 mA cm^−2^ current density. Such performance was significantly superior to that of 20% Pt/C, MoS_2_ NS/CF, and GDY/CF catalysts, as presented in Fig. 6f. Moreover, the hybridized GDY-MoS_2_ NS/CF exhibited the apparently smaller Tafel slope of 87.5 mV dec^−1^ than the MoS_2_ NS/CF and GDY/CF (Fig. 6g). Under the visible light irradiation, the photocurrent density of GDY-MoS_2_ NS/CF hybrid reached to 102 μA cm^−2^, which was approximately 46 times higher than that of pristine MoS_2_ NS/CF, further verifying the expected recombination suppression of the photogenerated charge carriers. In such GDY-MoS_2_ NS/CF structure, the electron-rich GDY not only provides more active sites to anchor active materials in the electrolyte, but also facilitates the photogenerated charge transport, thanks to excellent charge carrier mobility and electric conductivity of GDY, thereby improving HER activity.

### MXenes

Among various 2D materials, metal-free GCN and TMDs materials show high potential for fabricating high-quality photoelectrodes, while new layered MXene structures have recently attracted strong interest for the assembly of PEC system for water splitting. MXenes, a new and promising class of over 60 kinds of 2D metal carbides, nitrides or carbonitrides, is utilized to construct various electrodes applied in supercapacitors, photoelectrocatalysis, and photovoltaics [112–114]. In the past decade, MXenes have demonstrated many unique advantages to improve the PEC efficiency. For example, numerous unsaturated metal sites (such as Ti, Nb or V) are exposed due to the formation of 2D layer structures, which can result in the high redox reactivity compared to the single elemental carbon materials. Meanwhile, hydrophilic functionalities (such as -OH and -O) are dangled on the surface of MXenes, which is beneficial to bond with the diverse device components, such as semiconductors, as well as to stabilize 2D MXenes in aqueous solution over a long term. On the other hand, similar to graphene, MXene has excellent metallic conductivity, which also helps ensure the efficient charge transfer. With the above outstanding properties of the MXene family, 2D MXenes represent promising materials to construct the efficient photoelectrodes [115]. Similar to graphene, from the top view, monolayer MXene possesses a hexagonal lattice with a rhombohedral unit cell. From the side view, tri-layer sheets contribute to monolayer MXene, including two M transition metal layers sandwiching one X layer, which can function as active catalytic sites for HER. Nevertheless, MXene materials working as HER electrode require a high overpotential for water splitting [116]. Therefore, integrating MXene photoelectrode with new functional components is a feasible approach to overcome this weakness.

**Fig. 7:**
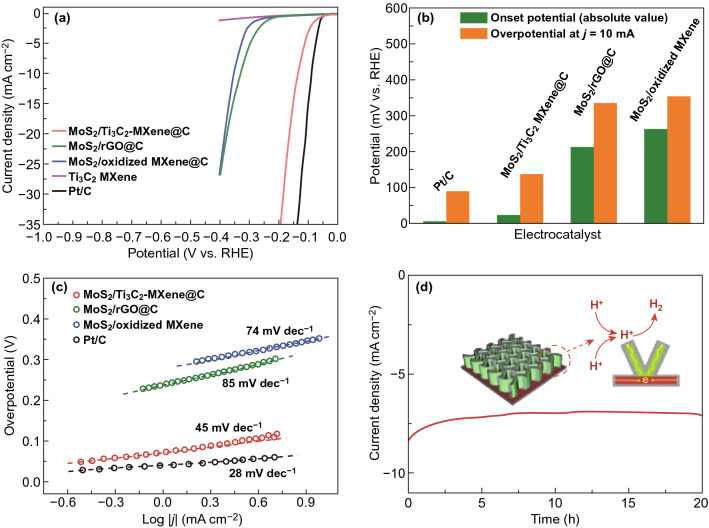
**a** Polarization curves of MoS_2_/Ti_3_C_2_-MXene@C, MoS_2_/rGO@C, MoS_2_/oxidized MXene, Ti_3_C_2_ MXene, and Pt/C in 0.5 M H_2_SO_4_, **b** onset potential and overpotential, and **c** Tafel plots of MoS_2_/Ti_3_C_2_-MXene@C, MoS_2_/rGO@C, MoS_2_/oxidized MXene, and Pt/C, **d** time-dependent current density curves with overpotential of 130 mV for MoS_2_/Ti_3_C_2_-MXene@C. Reproduced with permission from Ref. [119]. Copyright 2017 WILEY–VCH

Recently, nanocarbons have been encapsulated into MXene-based systems for promoting strong interfacial coupling and improving the separation and injection efficiency of photogenerated charge carriers [117, 118]. For instance, Qiu’s group took advantage of the carbon encapsulation route to not only stabilize the 2D Ti_3_C_2_-MXene material, but also to construct a hierarchical MoS_2_/Ti_3_C_2_-MXene@C heterojunction, which exhibited excellent HER performance and structural stability [119]. A small overpotential of 135 mV at 10 mA cm^−2^ was achieved using MoS_2_/Ti_3_C_2_-MXene@C catalyst in a 0.5 M H_2_SO_4_ solution, as shown in Fig. 7a, b, while the onset potential was around − 20 mV toward HER, which is close to commercial Pt/C. Meanwhile, Fig. 7c reveals that the Tafel slope of MoS_2_/Ti_3_C_2_-MXene@C significantly decreased to 45 mV dec^−1^ in comparison with the Ti_3_C_2_-MXene alone, while it was still inferior to conventional Pt/C catalyst with 28 mV dec^−1^. As shown in Fig. 7d, the MoS_2_/Ti_3_C_2_-MXene@C exhibited a stable current density at a constant potential of -130 mV for a long period of 20 h. Besides, Zhang et al. fabricated a cobalt-topped CNT/Ti_3_C_2_ nanosheet by ZIF-67 transformation [120]. The ZIF-67 as carbon source was transformed to the Co-CNTs on the Ti_3_C_2_ nanosheets via a pyrolysis process, where the layered Ti_3_C_2_ worked as conductive scaffolds to support the growth of Co-CNTs. Consequently, the Co-CNT/Ti_3_C_2_ exhibited a competitive electrocatalytic activity with the enhanced stability with respect to commercial Pt/C. One can attribute these achievements to the rich Co–N/C active sites, high graphitization, and large surface area.

These results indicated that 2D MXene/nanocarbons are indeed among the promising candidates as photocathodes in PEC system for the efficient renewable energy conversion. However, further improvements in PEC-HER activities over 2D MXene/nanocarbons photocathodes are required because 2D MXenes still do not present the expected photoresponse to produce photoinduced electrons and holes, which is important for solar-driven PEC-HER. Therefore, photoactive components should be introduced to form triadic photocathodes by coupling with 2D MXene/nanocarbons as co-catalysts due to their superior metal conductivity. The representative GCN-, TMDs- and LBO-based 2D photocathodes with nanocarbons for PEC-HER are summarized in Table 1.

**Table 1:** Comparison of PEC-HER activity of various 2D photocathodes

Photocathodes	Synthesis method	Electrolyte	*J*	IPCE	References
g-C_3_N_4_/carbon dots/PET	Hydrothermal method and electrophoretic deposition	5 v/v % triethanolamine (TEOA) and 0.5 M Na_2_SO_4_, pH = 10.5	38 μA cm^−2^ at 1 V vs. RHE	6.5% at 400 nm	[85]
CN-RGO	Calcination	10% (v/v) TEOA and 0.1 M KOH	~0.12 mA cm^−2^ at 1.23 V vs. RHE	5.3% at 400 nm	[86]
g-C_3_N_4_/RGO/Ni foam	Hydrothermal and electrophoretic deposition	0.5 M NaOH, pH = 6.8	~0.5 mA cm^−2^ at 0.4 vs. SCE	–	[87]
N-cGO/MoS_2_	Aerosol & calcination	0.5 M H_2_SO_4_	~0.14 mA cm^−2^ at -0.1 V vs. RHE	–	[105]
MoS_2_/CdS/rGO	Hydrothermal	0.35 M Na_2_SO_3_ & 0.25 M Na_2_S, pH = 12.5	~20.2 mA cm^−2^ at -0.6 V vs. RHE	–	[106]
GDY-MoS_2_ nanosheets/CF	Hydrothermal and deposition	1.0 M KOH	10 mA cm^−2^ at 0.09 V vs. RHE	–	[111]
Mo_2_C/graphene	Chemical vapor deposition	0.5 M H_2_SO_4_	10 mA cm^−2^ at -0.25 V vs. RHE	–	[183]
MoS_2_/Ti_3_C_2_@C	Etching and annealing	0.5 M H_2_SO_4_	10 mA cm^−2^ at -0.135 V vs. RHE	–	[119]

### Layered Double Hydroxides (LDHs)

The above-discussed GCN and TMDs materials are mainly utilized to assemble 2D photocathodes for PEC-HER. Correspondingly, the efficient 2D photoanodes for the PEC-OER are fabricated by using LDHs [121–123] and metal oxides [124–126]. Particularly, as a typical 2D material, LDHs have a layered stacking structure, where six oxygen atoms are situated at six corners and one transition metal atom is located at the center of an octahedron, denoted as MO_6_. These octahedrons further form a 2D layered structure by sharing the edge atoms. In general, the LDHs materials can be described by using a common chemical formula of M_1−*x*_^II^M_*x*_^III^(OH)_2_^x+^(A^n−^)_*x*/n_·mH_2_O with brucite-like M^II^(OH)_2_ layers. Some of the M^II^ cations can be replaced by M^III^ cations, leading to the formation of positively charged layers; thus, more anions are subsequently required to maintain the charge balance. Monovalent cations (e.g., Li^+^), divalent cations (e.g., Fe^2+^, Ni^2+^) [127–129], or trivalent cations (e.g., Co^3+^, Ti^3+^) [130–132] are often formed by constructing the positively charged layers with partial cation substitution. Accordingly, other anions including NO_3_^−^, SO_4_^2−^, and Br^−^ are often used to replace the original intercalation CO_3_^2−^ anion. The customized 2D nanosheets can form active sites exposed fully, thus optimizing the catalytic performance in various reactions [133–135]. The LDHs have attracted strong recent interest for OER catalysis under light irradiation [136–138]. It was reported that the 2D LDHs materials have numerous apparent advantages, for example, large surface area, abundant active sites, controllable layered structures, tunable chemical composition by varying the cations ratio, stable structure, and hierarchical porosity that is beneficial for water molecule diffusion and product release [139, 140]. Furthermore, the strong electrostatic interactions between the anion and cation layers endow the LDHs materials with ordered arrangement of interlayer species and tailorable orientation of active sites, which accelerates the movement of photogenerated charge carriers, thus boosting the OER activity [141].

For pristine LDHs photoanodes, since the OER performance is still low in comparison with traditional metal oxide photoanodes, the introduction of a new constituent is necessary [142, 143]. One of the feasible approaches is to combine with nanocarbon co-catalysts, such as 2D graphene and GDY, 1D CNTs or 0D CDs. With the above-discussed merits, nanocarbons as co-catalysts can not only offer electrically conductive pathways to LDHs but also gainfully enlarge the surface area for fast mass transfer, which is expected to significantly increase charge carriers separation, transfer, and injection efficiencies [144–146].

**Fig. 8:**
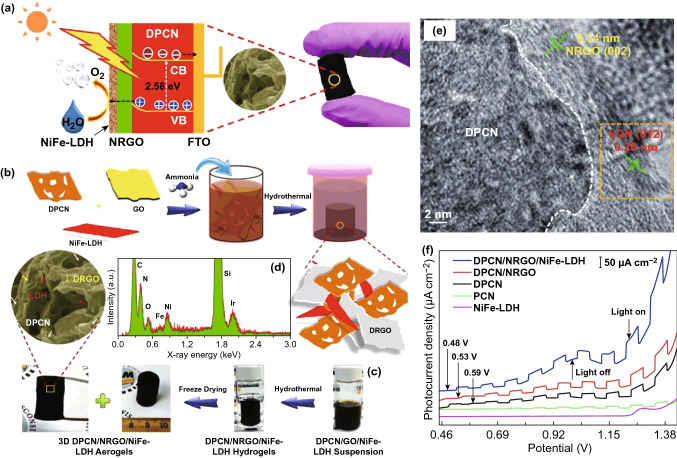
**a** Illustration of PEC mechanism over synthesized 2D photoanode, **b** schematic fabrication process of DPCN/NRGO/NiFe-LDH, **c** pictures of the obtained aerogel samples, **d** EDX spectrum, and **e** HRTEM image of DPCN/NRGO/NiFe-LDHs, **f** photocurrent density vs. applied potential for PCN, DPCN, NiFe-LDH, DPCN/NRGO, DPCN/NRGO/NiFe-LDH under chopped AM 1.5G irradiation. Reproduced with permission from Ref. [150]. Copyright 2016 American Chemical Society

Some research groups demonstrated that the coupling of LDH nanosheets with nanocarbons apparently decreased the onset potential with respect to commercial Ir/C catalyst and increased catalytic activity by providing the effective electrical pathway and high surface area [147–149]. Recently, Hou et al. developed a ternary N-deficient porous C_3_N_4_/N-doped graphene/NiFe-LDHs (DPCN/NRGO/NiFe-LDHs) aerogel photoanode consisting of N-deficient g-C_3_N_4_, N-doped graphene, and NiFe-LDHs by a facile hydrothermal route for the efficient PEC-OER, as shown in Fig. 8a–c [150]. The layered DPCN and NiFe-LDH nanosheets were firstly synthesized through the pyrolysis of urea and liquid exfoliation process, respectively. Subsequently, the resulting DPCN and NiFe-LDH were mixed with RGO via a self-assembly process under hydrothermal conditions to form the DPCN/NRGO/NiFe-LDH hybrid. The EDX spectroscopy demonstrated the C, N, Ni, Fe, and O are the principal elementals that exist in the DPCN/NRGO/NiFe-LDH structure, as presented in Fig. 8d. The HRTEM image further confirmed the intimate interfacial contacts among the three components shown in Fig. 8e. In this hybridized system, the introduced 3D N-doped graphene worked as an electron mediator to shuttle photogenerated charge carriers between N-deficient C_3_N_4_ and NiFe-LDHs, which resulted in the enhancement of separation and transfer efficiency of photogenerated charge carriers. The as-prepared DPCN/NRGO/NiFe-LDHs photoanode displayed an optimum configuration for PEC-OER by combining the advantages of each component with the merits of 3D aerogels. The photocurrent density of DPCN/NRGO/NiFe-LDHs reached 72.9 μA cm^−2^ at 1.22 V vs. RHE for OER under AM 1.5G irradiation, as presented in Fig. 8f, and the IPCE of 2.5% at 350 nm.

In order to further accelerate the interfacial mass and electron transport in photoelectrodes during PEC-OER process, more carbon-containing systems have been recently developed. For example, Wu and co-workers utilized GDY as an excellent electron mediator [151]. The superhydrophilic 2D GDY photoelectrode was synthesized through an air plasma route. Subsequently, the superhydrophilic GDY was electrostatically combined with ultrathin CoAl-LDH nanosheets. The 2D CoAl-LDH/GDY photoelectrode displayed an enhanced PEC-OER activity with an overpotential of 258 mV at 10 mA cm^−2^ and TOF of 0.60 s^−1^ at 300 mV. Meanwhile, the IPCE and photocurrent density of CoAl-LDH/GDY/BiVO_4_ hybrid reached the highest value, ~ 50% at 420 nm and ~ 3.15 mA cm^−2^ at 1.23 V vs. RHE, respectively. The half-cell photoconversion efficiency of superhydrophilic CoAl-LDH/GDY combined with BiVO_4_ was evaluated, displaying a significant increase up to 0.63%, by comparing with other GDY-free LDH-based BiVO_4_ photoanodes. Furthermore, DFT calculations indicated that the improvement in interfacial electron and mass transport was achieved through the utilization of superhydrophilic GDY, due to the strong interaction between GDY and CoAl-LDH, which is beneficial for absorbing water molecules around catalysts and boosting the PEC-OER activity.

**Fig. 9:**
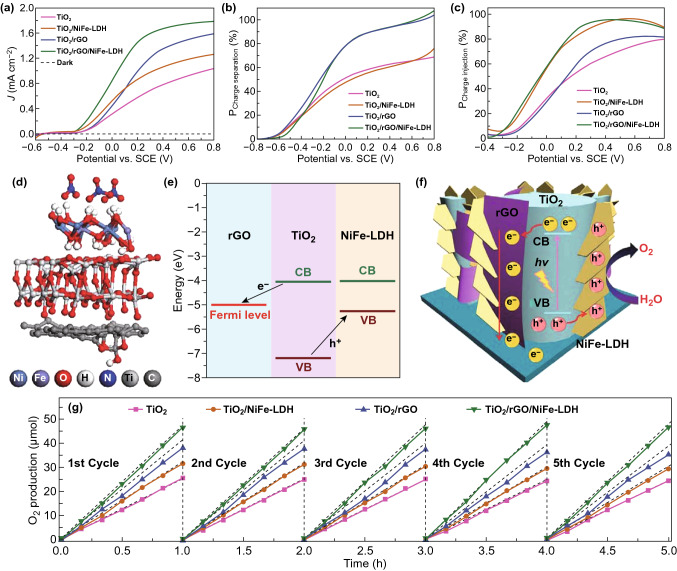
**a** Current–potential curves, **b** charge separation, and **c** injection efficiencies vs. potential of TiO_2_, TiO_2_/NiFe-LDH, TiO_2_/rGO, TiO_2_/rGO/NiFe-LDH photoanodes, **d** optimized geometries of TiO_2_/RGO/NiFe-LDH, **e** band alignments of TiO_2_, RGO, and NiFe-LDH, **f** scheme of water oxidation process in hybridized TiO_2_/rGO/NiFe-LDH photoanode, **g** actual O_2_ evolution of TiO_2_, TiO_2_/NiFe-LDH, TiO_2_/rGO, TiO_2_/rGO/NiFe-LDH (the dot lines denote the theoretical O_2_ generation estimated from the measured photocurrent with Faradaic efficiency of 100%). Reproduced with permission from Ref. [156]. Copyright 2016 Royal Society of Chemistry

Based on the optimum PEC configurations, the nanocarbons/LDHs systems as active components are further utilized to integrate with other semiconductors, such as TiO_2_, Cu_2_O, and BiVO_4_, for constructing high-quality three-component photoanodes [152–155]. For instance, Ning et al. reported a feasible strategy to construct a ternary hybridized PEC system by introducing RGO and NiFe-LDHs onto TiO_2_ nanorod arrays (NAs), which not only improved the separation and transport of the photogenerated charge carriers, but also increased the PEC-OER efficiency, as shown in Fig. 9e, f [156]. Firstly, TiO_2_ NAs were vertically grown on FTO substrate via a customized hydrothermal method, and then, layered RGO was deposited onto TiO_2_ NAs and annealed for strong adhesion. Subsequently, layered NiFe-LDH was uniformly electrodeposited on the surface of TiO_2_/RGO NAs to fabricate a ternary TiO_2_/rGO/NiFe-LDH photoanode. The combined experimental and computational studies revealed that the added RGO was able to efficiently collect the photogenerated electrons from TiO_2_ owing to its high work function and fast electron mobility, as shown in Fig. 9d. As a result, the electron transport was enhanced, and NiFe-LDH functioned as an effective OER electrocatalyst.

Owing to this synergistic effect, the hybridized photoanode exhibited a significantly improved photocurrent density of 1.74 mA cm^−2^ at 0.6 V and photoconversion efficiency of 0.58% at 0.13 V, which was superior to that of the TiO_2_-based photoanodes, as shown in Fig. 9a. Meanwhile, the estimated charge carrier separation and injection efficiencies of the hybridized system are presented in Fig. 9b, c. It was found that charge carrier separation efficiency of 98% at 0.6 V on TiO_2_/RGO/NiFe-LDH NAs was significantly enhanced as compared with 66% at 0.6 V on pristine TiO_2_ NAs. Meanwhile, the charge injection efficiency showed an apparent improvement for TiO_2_/RGO/NiFe-LDH, achieving 95%, with respect to 76% of bare TiO_2_ NAs, inferring a highly efficient PEC-OER activity. Moreover, ternary hybridized photoanode exhibited an average O_2_ evolution yield of 15.5 mmol h^−1^ cm^−2^ with the average Faradaic efficiency of 97%, as displayed in Fig. 9g, which was 1.88 times higher than that of pristine TiO_2_ NAs.

This result further confirmed that the introduction of RGO and NiFe-LDH apparently promoted the PEC-OER activity with considerable stability under sunlight irradiation, where the photoinduced electrons transfer to the RGO from the conduction band of TiO_2_ and the holes to NiFe-LDH from the valence band of TiO_2_. Based on this effective strategy, Xiang and co-workers also reported an integration of rGO-LDH-BiVO_4_ by assembly of 2D BiVO_4_ photoanode with CoAl-LDHs and graphene [157]. A remarkable improvement in the PEC-OER performance was obtained by this hybridized photoanode, compared with CoAl-LDH-BiVO_4_ and bare BiVO_4_ cases. The photocurrent density and IPCE of rGO-LDH-BiVO_4_ photoanode reached 2.13 mA cm^−2^ at 1.23 V vs. RHE and 52% at 400 nm, which were 4.0 and 2.5 times higher compared to the pristine BiVO_4_ photoanode.

These results revealed that the enhancement of charge carriers separation efficiency and water oxidation kinetics could be mainly attributed to the introduction of rGO and CoAl-LDH. Similar as the above-mentioned TiO_2_/RGO/NiFe-LDH photoanode, such charge transfer pathways significantly shorten the carrier transportation distance and effectively suppress the carrier recombination. Consequently, the PEC-OER efficiency of the photoanode can be apparently enhanced by designing this triadic heterostructure.

On the other hand, CDs were used to substitute graphene to construct carbon-based NiFe-LDHs@BiVO_4_ photoanode [158]. The introduction of CDs not only reduced the charge transfer resistance, but also lowered the overpotential for OER catalysis. As a result, ternary hybridized 2D photoanode exhibited the significantly enhanced photocurrent and IPCE value compared with the NiFe-LDH/BiVO_4_. The IPCEs measured at 380 nm for BiVO_4_, CDs/BiVO_4_, NiFe-LDH/BiVO_4_, and CDs/NiFe-LDH/BiVO_4_ were 19.17%, 22.97%, 24.59%, and 40.94%, respectively. Meanwhile, the CDs/NiFe-LDH/BiVO_4_ hybridized photoanode had the highest conversion efficiency of 0.58% at 0.82 V, outperforming the NiFe-LDH/BiVO_4_ catalyst with 0.34% at 0.91 V, CDs/BiVO_4_ with 0.17% at 0.99 V, and pristine BiVO_4_ with 0.09% at 0.96 V. Although the charge separation efficiency of 66.7% at 1.23 V for CDs/NiFe-LDH/BiVO_4_ had no noticeable improvement compared to other photoanodes, the charge injection efficiency was notably enhanced from 69.2 to 92.8% at 1.23 V.

These data clearly suggest that the improved catalytic activity was due to the reduced OER overpotential and the enhanced charge transport kinetics. When the photoinduced holes accumulate at the surface of LDHs, the overpotential of OER at the solid–liquid interface depends on the energy barrier and reaction rate. As a result, the decreased OER overpotential could significantly improve the PER-OER activities.

### Layered Bismuth Oxyhalides (LBOs)

Apart from the 2D LDHs for PEC-OER catalysis, most of the photoanodes have been made of semiconducting metal oxides due to the apparent chemical stability and facile fabrication processes, which are important factors for practical large-scale applications. Therefore, 2D metal oxides with the broad photoresponse spectrum and high absorption efficiency are typically chosen as light absorbers to optimize the PEC water splitting. Among various semiconducting metal oxides, ternary metal oxides attract more and more attention as efficient photoanodes involved in a PEC system for efficient OER [159].

Commonly, ternary metal oxide is often defined as an oxide matrix consisting of two different metal ions. Compared with binary oxides, such as TiO_2_ [160], ZnO [161], and WO_3_ [162, 163], ternary metal oxides possess rich and tunable compositions, which leads to controllable atomic and electronic structures as photoelectrodes with tunable light absorption [164, 165]. In previous reviews [166–168], ternary BiVO_4_-related photoelectrodes have been summarized and discussed; hence, 2D BiVO_4_ as a common supporting material in PEC systems is not discussed further.

**Fig. 10:**
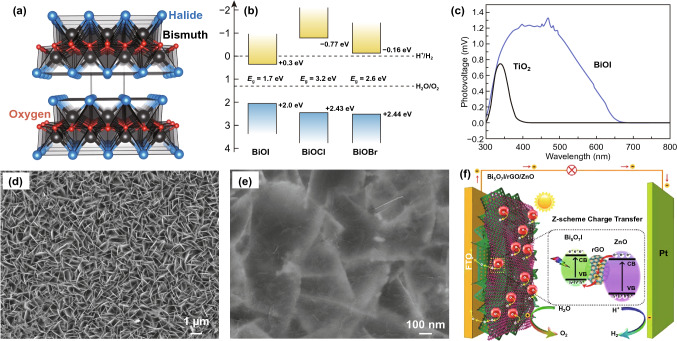
**a** Crystal structure of bismuth oxyhalide systems, **b** band alignments of BiOX at pH = 0, **c** surface photovoltaic spectroscopies of BiOI and TiO_2_, **d, e** SEM images of porous Bi_5_O_7_I and Bi_5_O_7_I/rGO/ZnO, **f** scheme of charge transfer mechanism of Bi_5_O_7_I/rGO/ZnO. Reproduced with permission from Ref. [173, 175, 179]. Copyright 2016 Royal Society of Chemistry, Copyright 2009 and 2019 Elsevier

Compared with conventional BiVO_4_-based photoanodes, ternary layered bismuth oxyhalides have variable layer thickness, suitable band alignments accompanied with tunable chemical compositions, and highly exposed dangling bonds as active sites for water splitting [169–171]. Generally, layered bismuth oxyhalides, BiOX (X = Cl, Br, I), are a family of ternary V-VI-VII semiconductors with a tetragonal matlockite crystal structure and atomic composition-related bandgap energy, where a layered nanostructure is constituted by stacked [X-Bi-O-Bi-X] slabs via van der Waals interactions, as shown in Fig. 10a, b [172, 173]. In this model, one bismuth atom is surrounded by four oxygen atoms and four halogen atoms, forming an asymmetric decahedral. The interlayer van der Waals attraction arising through the strong covalent bonding between the layers creates more interesting properties in the fields of anisotropic structures, electrics, and optics [174]. Zhang and co-workers reported BiOI nanoplates as a new 2D material for assembling solar cells by encapsulating BiOI into chitosan, which displayed a promising PEC activity, as shown in Fig. 10c [175]. Since then, layered BiOI and similar materials have been widely used in photoelectrodes for water splitting and environmental remediation [176–178].

To further enhance the PEC performance of layered bismuth oxyhalides-based photoelectrodes, nanocarbons have been utilized to enhance the conductivity, which is beneficial for fast movement of photogenerated charge carriers and effective suppression of recombination. For example, Jiao et al. used bismuth oxyiodide to assemble the Bi_5_O_7_I nanosheets/RGO/ZnO heterojunction by depositing ZnO QDs/RGO on the surface of Bi_5_O_7_I nanosheets (Fig. 10f) [179]. Firstly, porous Bi_5_O_7_I nanosheets enriched with O vacancies were prepared through a reductive calcination of BiOI (Fig. 10d), which facilitated light harvesting as well as separation and transportation of photogenerated charge carriers. Further, ZnO QDs/RGO was deposited on the 2D Bi_5_O_7_I photoanode to form a heterojunction, as presented in Fig. 10e. Under light illumination, the photoinduced electrons on the conduction band of ZnO could transfer to the valence band of Bi_5_O_7_I via RGO nanosheets, thus leading to the quenching of photoexcited holes at Bi_5_O_7_I. Therefore, the effects not only help to improve the efficient separation of photogenerated electron–hole pairs, but also maintain the original excellent PEC redox ability. Consequently, a high PEC-OER performance was achieved over Bi_5_O_7_I/RGO/ZnO heterojunction with photocurrent density of 15 μA cm^−2^, which was the highest value among all the prepared Bi_5_O_7_I-based samples.

We articulate that the studies on integration of 2D BiOX photoelectrodes with nanocarbons for PEC water splitting are still at initial stage. In the future, the investigation on utilizing nanocarbons to modify 2D BiOX is necessary for constructing 2D integrated photoelectrodes. At the same time, various design strategies regrading solar-driven photocatalysts, such as tuning localized surface nanostructures [180], exposing efficient surfaces of photogenerated charge carriers transfer [181], and introducing desirable components [182] in 2D BiOX photoelectrodes, may offer new paths to improve charge carrier transport between the nanocarbons and 2D BiOX, reduce redox HER/OER potentials, thereby further increasing the solar harvesting efficiency. These representative LDHs- and LBO-based 2D photoanodes with nanocarbons for PEC-OER are summarized in Table 2.

**Table 2:** Comparison of PEC-OER activity of various 2D photoanodes

Photoanodes	Synthesis method	Electrolyte	*J*	IPCE	References
DPCN/NRGO/NiFe-LDH	Pyrolysis and hydrothermal	0.01 M Na_2_SO_4_, pH = 6.8	72.9 μA cm^−2^ at 1.22 V vs. RHE	2.5% at 350 nm	[150]
CoAl-LDH/GDY/BiVO_4_	Air plasma and wet deposition	0.1 M Na_2_SO_4_, pH = 6.8	3.15 mA cm^−2^ at 1.23 V vs. RHE	~50% at 420 nm	[151]
BiVO_4_/RGO/NiFe-LDH	Electrodeposition and annealing	0.1 M phosphate buffer saline, pH = 7	1.13 mA cm^−2^ at 1.23 V vs. RHE	51.08% at 420 nm	[152]
TiO_2_/RGO/NiFe-LDH	Hydrothermal and spin coating and electrochemical deposition	0.5 M Na_2_SO_4_, pH = 6.8	1.74 mA cm^−2^ at 1.25 V vs. RHE	5.12% at 400 nm	[156]
RGO/CoAl-LDH/BiVO_4_	Hydrothermal	0.1 M phosphate buffer saline, pH = 7	2.13 mA cm^−2^ at 1.23 V vs. RHE	52% at 400 nm	[157]
CDs/NiFe-LDH/BiVO_4_	Electrochemical deposition	0.5 M phosphate buffer saline, pH = 7	1.49 mA cm^−2^ at 1.23 V vs. RHE	40.94% at 380 nm	[158]
Bi_5_O_7_I/RGO/ZnO	Annealing and wet deposition	0.1 MNa_2_SO_4_, pH = 6.8	0.21 mA cm^−2^ at 1 V vs. SCE	–	[179]

## Conclusion and Perspectives

In summary, we have critically reviewed recent developments regarding rational PEC configurations consisting of photoelectrodes, interlayers, and co-catalysts to achieve sunlight harvesting in a broad spectral range, efficient separation and transfer of photogenerated charge carriers, as well as rapid reaction kinetics for water splitting. To achieve all these criteria, elaborately designed 2D photoelectrodes have been widely utilized, including GCN, TMDs, and MXene for PEC-HER, LDHs and LBOs for PEC**-**OER. These materials are promising for solar-driven water splitting in PEC systems. Following the effective optimization principles, metal-free nanocarbons, such as 0D CDs, 1D CNTs, 2D graphene, and 2D GDYs, have been used as electron mediators to improve the separation and transport efficiencies of photogenerated charge carriers in high-performance PEC systems for water splitting. Meanwhile, the state of the art in the synthesis and characterizations of 2D photoelectrodes hybridized with nanocarbons for superior efficiencies in PEC water splitting has been critically reviewed.

In this context, GCN material is widely studied as a 2D photoelectrode due to its unique layered structure, suitable bandgap, and a suitable negative conduction band for HER. In this application, the GCN not only acts as a supporting material to harvest solar energy, but also functions as an effective electrocatalyst to drive electrocatalytic HER. To improve PEC-HER performance, 2D graphene and 0D CDs are frequently introduced into the GCN to enhance the separation and transport efficiency of photogenerated charge carriers. Similar to 2D GCN, when used alone, 2D TMD (especially MoS_2_) HER catalysts also face poor separation and severe recombination of photogenerated charge carriers.

To overcome these problems, a series of nanocarbons, such as graphene or CNTs, with high charge mobility as co-catalysts have been introduced to assemble 2D photocathodes, which exhibit a significant enhancement in PEC-HER performance. Furthermore, although the emerging 2D MXenes possesses metallic conductivities and highly exposed metal sites, which present the strong redox activity for HER, the separation and transport efficiencies of the photogenerated charge carriers still require substantial improvement. Therefore, nanocarbons, such as CNTs, as co-catalysts and electron mediators are introduced into 2D MXenes to promote the charge carrier separation and transfer in 2D photocathodes for the enhanced PEC-HER activity under light illumination.

Apart from the above-mentioned 2D GCN, TMDs, and MXenes for efficient HER, 2D LDHs have been explored as Ru or Ir-free photoelectrodes for obtaining high PEC-OER activity because the LDHs materials contain transition metals of Fe, Co, and Ni. These metals facilitate PEC-OER with relatively low overpotentials, thereby accelerating water splitting. Importantly, 2D LDH materials possess tunable chemical composition by varying the ratios of different cations, porous nanostructures with large specific surface area, highly exposed active sites with high catalytic reaction rates, as well as high structural stability. Based on these merits, diverse LDHs-based 2D photoanodes utilizing nanocarbons have been analyzed, which show significant performance improvement in PEC water splitting in comparison with bare LDHs photoanodes.

Meanwhile, bismuth oxyhalides (e.g., BiOI) are often used as effective light absorbers for PEC-OER. Since semiconducting bismuth oxyhalides have a sufficiently positive valence band, the photoinduced holes with strong oxidative abilities can effectively oxidize water to produce O_2_. Therefore, these bismuth oxyhalides are combined with layered nanocarbons (e.g., graphene and GDY) as co-catalysts to form hybridized photoanodes, eventually achieving the expected improvement in the PEC-OER performance.

Although remarkable advances have been made to enhance the PEC-HER and PEC-OER performances by coupling 2D photoelectrodes with nanocarbon co-catalysts, up to now, the roles of 2D support materials and nanocarbons in heterostructured photoelectrodes are not fully understood due to the lack of strong experimental evidences and sufficient theoretical insights. Therefore, comprehensive understanding of the reactive mechanisms is a viable path to design and fabricate high-performance 2D integrated photoelectrodes for water splitting. Besides, the charge carrier dynamics in PEC system should be further uncovered based on a series of PEC tools, such as IMPS/IMVS, TAS, TRPL, and EIS. Meanwhile, a combination of advanced in situ techniques, for instance, in situ XPS, in situ TEM, and in situ X-ray absorption spectrum (XAS), should be used to reveal correlations between structure and PEC performance.

On the other hand, due to the limitations of the available experimental technologies, there is an urgent need to develop reliable theoretical models and simulations to explain the optimized PEC system with 2D photoelectrodes by utilizing artificial intelligence, high-performance computing, and/or big data analytics. Meanwhile, it is useful to further advance the visualization of photogenerated charge carriers inside 2D photoelectrodes based on simulation software and scanning tunneling microscope (STM) and further optimize the configuration of PEC systems.

Nowadays, direct comparisons of PEC-HER/OER performances among the many different nanocarbons-enhanced photoelectrodes are quite challenging because of the different compositions, amounts, and test parameters, which significantly affect the PEC results. It is required that the test principles and conditions of PEC-HER/OER should be unified in the future. Furthermore, the fabrication procedures of 2D photoelectrodes and nanocarbons are the key determinants of the final water splitting efficiencies. Therefore, it is necessary to develop several feasible and unified synthetic protocols, such as electrochemical or hydrothermal routes for better comparison and finding the optimal material system. This approach is promising to translate the laboratory-scale research into the widespread commercial applications of the PEC-HER and PEC-OER processes.
